# Tolerance of Violence against Women and the Risk of Psychosocial Distress in Humanitarian Settings in Northern Uganda

**DOI:** 10.3390/ijerph18158103

**Published:** 2021-07-30

**Authors:** Paul Bukuluki, Peter Kisaakye, Bonny Etti, Micheal Ocircan, Roberts-Reites Bev

**Affiliations:** 1 School of Social Sciences, Makerere University, Kampala 7062, Uganda; 2 School of Statistics and Planning, Makerere University, Kampala 7062, Uganda; pkisaakye@gmail.com; 3 Save the Children, Kampala 12018, Uganda; bonny.etti@savethechildren.org (B.E.); Michael.ocircan@savethechildren.org (M.O.); bev.roberts-reites@savethechildren.org (R.-R.B.)

**Keywords:** violence against women, psychosocial distress, humanitarian settings, refugees, host communities, Northern Uganda

## Abstract

Background: Violence against women (VAW) remains a public health concern, which can sometimes lead to mental or psychological stress among other negative consequences. Data and methods: we fitted a binary logistic regression model on 657 respondents from host and refugee settings in three humanitarian districts (Adjumani, Obongi, and Lamwo) to examine the determinants of psychosocial stress. Results: experience of psychosocial distress is higher among refugees than host populations. Results indicate a higher proportion of respondents who ever experienced psychosocial stress in the 6 months preceding the survey among those who believed that a woman should tolerate violence (59% vs. 53%). Respondents who believed that a woman should tolerate violence had higher odds of experiencing psychosocial stress than their counterparts who believed a woman should not tolerate violence (OR = 6.86; 95%CI = 1.23–38.22). The likelihood to experience psychosocial stress was higher among females (OR = 6.94; 95%CI = 1.76–27.32), those with primary education (OR = 4.73; 95%CI = 1.24–18.00), and respondents with less than USD 2.7 as personal income one month before the survey (OR = 3.37; 95%CI = 1.32–8.62). Respondents who said that women should engage in income generation activities had higher odds to experience psychosocial stress (OR = 0.39; 95%CI = 0.17–0.89). Conclusion: results suggest that income and positive attitudes toward female-led income generating activities act as protective measures against psychosocial distress. Given the associations between VAW and psychosocial distress, efforts aimed at prevention and response to VAW in humanitarian settings should integrate mental health and psychosocial support interventions.

## 1. Introduction

Violence in all its forms remains a public health concern across the world [1,2]. Intimate partner violence (IPV) can be physical, sexual, emotional, psychological, among other forms [3]. Previous research has shown that women are susceptible to poor health outcomes due to violence [3,4]. Violence against women can have direct health consequences related to injury or psychosocial distress, such as anxiety, depression, and trauma, stress—which may last longer even after violence is no more [4,5,6]. Such findings indicate that experience of psychosocial distress is higher among people who experience violence than people who do not experience violence. Previous research shows that women who are exposed to intimate partner violence are more likely to acquire HIV than women who have not experienced intimate partner violence [7]. Other negative health effects associated with violence include induced abortion, low birth weight, suicide, homicides, and alcohol disorders [8]. Due to this, there is a need to start recognizing violence against women as a societal or community problem rather than an individual problem [9].

While violence can occur in all settings, humanitarian settings that are associated with unstable relations, such as conflict displacements, often present ground for persistent violence [10]. Violence against women remains a significant problem in humanitarian settings [11,12,13], leading to negative psychosocial and health conditions [14]. The conditions present in humanitarian settings that are as a result of war or transition [15,16], poor economic living conditions [17], or limited social support [18] may facilitate occurrence of violence against women [19]. For example, the districts under study (Adjumani, Lamwo and Obongi) are located in the greater northern part of Uganda, which, for a long time, experienced a guerilla war (during the period 1986–2006) [20,21,22]. Humanitarian settings in Uganda were reported to be associated with a high prevalence of violence [23]. For example, the United Nations High Commissioner for Refugees (UNHCR) registered an estimated 4297 cases of gender-based violence in 12 refugee settlements between January and November 2019 [24,25].

Research shows that experience of psychosocial distress varies among different socioeconomic backgrounds. For example, previous research has pointed to a higher likelihood of experiencing psychosocial distress among the poor [26,27], women and girls [28], youth, refugees, those living in rural areas, people with low levels of education [15], substance or alcohol abusers [29], and female sex workers [30].

Despite a large body of evidence on the relationship between violence and psychosocial health, there is still a considerable gap in the understanding of the concept, especially among refugees in low- and middle-income countries [3,31,32].

This study therefore focuses on humanitarian settings in three districts (Adjumani, Lamwo, and Obongi) with a high occurrence of violence against women, but with limited prevention and intervention strategies [16,33], and high rates of gender inequality [34]. The three districts were included in the study because they are host to a large number of South Sudanese refugees, constituting one of the largest segments of refugees in Uganda [35,36], and share a similar culture [37].

Despite a large body of evidence on the relationship between violence and psychosocial health, there is still a considerable gap in the understanding of the concept, especially among refugees in low- and middle-income countries [3,31,32]. Therefore, the overall goal of this study was to increase understanding of the relationship between violence against women and psychosocial distress in humanitarian settings. This aligns with the resolution set by the United Nations General Assembly in 1993 to eliminate all forms of discrimination against women [38], and the international consensus statement on women’s mental health and interpersonal violence against women [39]. The findings in this study can help shed light on understanding the intersections between violence against women and mental health, especially psychosocial distress, particularly in humanitarian settings in low income countries. Based on this, we draw two hypotheses. First, the experience of psychosocial distress is higher among respondents who accept violence than respondents who do not accept violence. Second, the experience of psychosocial distress is higher among refugees than host communities.

## 2. Materials and Methods

### 2.1. Study Design and Approach

This study uses data from a cross-sectional survey to examine whether acceptability of violence against women influences the risk of experiencing psychosocial distress. The project’s aim is to contribute to strengthening the ongoing refugee response by focusing on the socioeconomic gender equality in the context of livelihoods and enhanced capacities for mainstreaming gender-based violence. Responses were from 657 respondents in three humanitarian districts (Adjumani, Obongi, and Lamwo) living in both host and refugee settlements. Adjumani and Obongi districts are located in the West Nile sub-region in the northern region, while Lamwo district is located in the Acholi sub-region in the northern region of Uganda.

### 2.2. Sampling

A sampling frame of all the Save the Children beneficiary individuals who were 18 years and older, from households, was developed, using lists provided by the camp commandant, under the Office of the Prime Minister (OPM) and local council authorities in charge of the respective study sites. All individuals who participated in the study were selected using systematic random sampling. We used Cochran’s formula [40,41,42] to estimate a sample size of 666 individuals out of the 2700 Save the Children project beneficiaries. The response rate was 99% since the actual sample size was 657 respondents from all study districts: Adjumani (223), Lamwo (218), and Obongi (216), as shown in Table 1.

### 2.3. Data Collection

Computer assisted personal interviewing (CAPI) technology was used to collect data. Research assistants who collected the data were trained for five days. A pre-test prior to the actual data collection exercise was conducted from the 5–6 June 2020, and any lessons learned from the pre-test were incorporated in the design of the final questionnaire. The main data collection exercise started on the 10 June until 26 June 2020. all standard operating procedures (SOPs) for collecting data during the COVID-19 pandemic, as guided by the World Health Organization, were adhered to [43].

### 2.4. Variables

The main independent variable was tolerance of violence against women or girls. Respondents were asked whether women should tolerate violence against women, to measure tolerance of violence against women. Responses to this question were either ‘No’ or ‘Yes’.

Information on age (18 years and older) was collected in single years. The survey collected information on the sex of the respondents (female or male), current marital status (married, divorced, separated, widowed, or single), level of education (no education, primary, secondary, tertiary or university), personal income in the last one month preceding the survey: less than UGX 10,000 (less than USD 2.7), UGX 10,000–20,000 (USD 2.7–USD 5.5) or above UGX 20,000 (above USD 5.5), whether women should participate in income generation activities (no or yes), whether consent should be sought before sex (no, do not know, or yes), “it is possible for men to stop violence” (agree, disagree, or do not know), “women can decide on the number of children they can have” (agree, disagree, or do not know), “women know where to obtain modern contraception” (yes, no, or do not know), and “women are raped because of the way they are dressed” (agree, disagree, or do not know). Age was categorized into four 10-year age groups (15–24, 25–34, 35–44, and 45–54 years) and an open-ended age group (55+) because of fewer cases above the age of 54 years. Respondents with tertiary or university education were lumped together because of fewer cases in each of the categories.

Following standard questions from the Kessler’s scale [44], respondents were asked whether they had ever experienced anxiety, panic, sadness, isolation, helplessness, withdrawal from others, a reduction in their display of emotion, felt physically weak, or experienced hallucinations or apathy in the 6 months preceding the survey. Responses to each question was either a ‘Yes’ or ‘No’. Respondents who experienced any of the above in the 6 months before the survey were categorized under ‘Yes’ and respondents who never experienced any of the above were categorized under ‘No’. The emerging composite variable was used to measure psychosocial distress.

### 2.5. Data Analysis

Data analysis was done using STATA software version 15.0 [45] to present frequency distributions, bivariate relationships, and multivariate results. We fitted a complementary log–log regression model to examine the risk of experiencing psychosocial distress. The first model controlled for only tolerance of violence against women or girls and the second model controlled for all variables that were significant at the bivariate level of analysis, apart from demographic variables.

### 2.6. Ethical Considerations

We sought permission to access the study sites from the Office of the Prime Minister (OPM) and Chief Administrative Officers (CAO). Permission to conduct the study was obtained from the School of Social Sciences at Makerere University. We obtained verbal informed consent from each respondent before the interview could start. Respondents were assured of confidentiality, privacy, voluntary participation, and were free to answer only what they were willing to answer or face no consequences for withdrawing from the study. To ensure privacy and confidentiality, research assistants were gender-matched. Interviews were conducted in a private place that was chosen or preferred by the respondent.

## 3. Results

### 3.1. Distribution of Respondents

Table 2 shows the distribution of respondents by resident status. Respondents were significantly different by resident status, by age of the respondent (*p* < 0.05), level of education (*p* < 0.05), personal income in the last month (*p* < 0.001), “women should participate in income generation” (*p* < 0.05), “women know where to obtain contraceptives” (*p* < 0.01), and study district (*p* < 0.001). Results in Table 2 show that, irrespective of resident status, the largest age category of respondents was 55 years or above, currently married, and female. Results also show that the majority had primary education, earned less than USD 2.7 in the previous month before the survey, agreed that women should participate in income generation activities, and that it was possible for men to stop violence. Further, the results in Table 2 indicate that, irrespective of resident status, the majority of respondents agreed that consent before sex is necessary and women know where to obtain contraceptives.

### 3.2. Tolerance of Violence against Women and Experience of Psychosocial Distress

**Figure 1:**
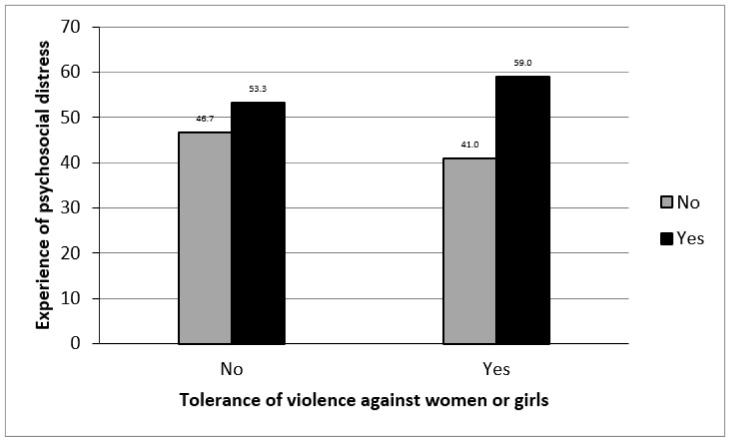
Tolerance of violence against women and experience of psychosocial distress.

Figure 1 shows the percentage distribution of respondents by tolerance of violence against women and psychosocial distress experience. The figure shows that the experience of psychosocial distress was highest among all respondents irrespective of whether they agreed or not to tolerance of violence against women or girls. However, the experience of psychosocial distress was observed to be higher among respondents who agreed to tolerance of violence against women or girls than those who did not (59% vs. 53%). The distribution shown in Figure 1 implies that while there are likely to be other factors that may be associated with psychosocial distress among all respondents, tolerance of violence against women or girls is an important factor.

### 3.3. Factors Associated with the Risk of Experiencing Psychosocial Distress

Table 3 shows that respondents who tolerated violence against women or girls had higher odds of experiencing psychosocial distress than their counterparts (OR = 6.86; CI = 1.23–38.22). Female respondents had higher odds than male respondents in regard to experiencing psychosocial distress (OR = 6.94; CI = 1.76–27.32). Respondents with primary education (OR = 4.73; CI = 1.24–18.00) had higher odds of experiencing psychosocial distress than respondents with no education. Respondents who earned less than USD 2.7 in the month preceding the survey had higher odds (OR = 3.37; CI = 1.32–8.62) of experiencing psychosocial distress than respondents who earned more than USD 5.5. However, the likelihood of experiencing psychosocial distress was less among respondents who said that women should participate in income generation (OR = 0.39; CI = 0.17–0.89) than their counterparts who said otherwise.

**Figure 2:**
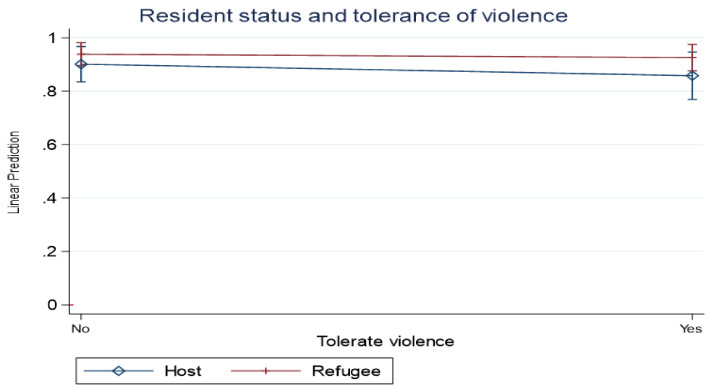
Marginal effects of resident status and tolerance of violence against women on experiences of psychosocial violence.

In order to better understand the interaction between resident status and tolerance of violence, we included an interaction term in the model. Using a graphical representation (it was suggested as being a better way to reflect the effects of interaction) [46], we show predictive margins of the interrelationship between tolerance of violence against women, residents status, and experience of psychosocial distress. Results in Figure 2 show that the experience of psychosocial distress was highest among refugees than host communities irrespective of tolerance of violence against women, although a widening gap was observed among respondents who tolerated violence against women. The results in Figure 2 imply that being a refugee or living in a refugee setting increases the risk of experiencing psychosocial distress, although tolerance of violence against women aggravates psychosocial distress.

## 4. Discussion

Tolerance of violence against women is an important risk factor for the physical and mental health of women [5,6]. The findings reported in this paper confirm the first hypothesis that individuals who accept violence are more likely to experience psychosocial distress than their counterparts who do not accept violence. When an interaction term between refugee status and tolerance of violence was introduced in the model, results indicated that experience of psychosocial distress was higher among refugees than host populations—confirming the second hypothesis.

Our results demonstrate that psychosocial distress is six times higher among respondents who have accepting attitudes towards violence against women compared to their counterparts. Tolerance of violence against women (by our respondents) needs to be put in context. Our respondents are born and socialized within patriarchal settings that are characterized by social and gender norms that embody hegemonic masculinity [47]. The expression of hegemonic masculinity tendencies in this case illustrates how gender inequality and oppression are embodied in relationships between men and women [48], in humanitarian settings that make both men and women normalize subordination of women through accepting attitudes and norms towards tolerance of violence. The unequal gender relations contribute to sustaining structures, i.e., perceiving hierarchical gender relations between men and women as normal and legitimate [47]. In this context, the power relations are skewed towards accepting violence against women as something normal and there are social sanctions that reward adherence to such social harmful gender norms, and retribution for those who try to exercise their agency to challenge the prevailing hegemonic social and gender norms that operate within the patriarchal systems and structures [49].

Our results are also in line with the social norms theory that conceptualizes acceptability of gender-based violence as embedded in the informal rules, which regulate the behavior of individuals in specific cultural contexts [50,51,52]. The associations between acceptability of violence against women and psychosocial distress points to the need for integrated programming around violence against women, intimate partner violence prevention and response, and psychosocial support interventions [53]. The link between harmful social norms and acceptability of violence points to the need to integrate mental health or psychosocial support programming with gender and social norm change interventions that aim to prevent gender-based violence or violence against women [50], particularly in humanitarian settings.

Results show that having a positive attitudes towards women’s empowerment, particularly through engaging in income generation activities, was associated with less likelihood of experiencing psychosocial stress. This implies that having a positive attitude towards women’s empowerment could have positive implications of psychosocial health and wellbeing. These findings are similar to studies that link women’s economic empowerment and the prevention of violence against women and intimate partner violence [27].

Results also demonstrate that the likelihood of experiencing psychosocial stress was higher among females, those with primary education, and respondents with low income (less than USD 2.7). This implies that having a low formal education (primary education) or a low income are risk factors for experiencing psychosocial distress. These results are similar to those of other studies that showed higher odds of experiencing psychosocial distress among the poor, women and girls, youth, refugees, those living in rural areas, and people with low levels of education [15,26,28]. The results imply that programs that aim to improve mental health and psychosocial wellbeing, and prevent gender-based violence, particularly in humanitarian settings, should integrate components of economic empowerment, increasing access to education, and should deliberately target women and girls as well as young people.

## 5. Limitations

There are two main limitations we highlight in this study. First, the data we used did not come from a population-based sample, but rather it was NGO beneficiary-based. Although the results reported in this study provide context, the conclusions generated are only generalizable to this population under study. Second, the results generated should be interpreted with caution, given the large confidence intervals brought about by a small sample size.

## 6. Conclusions

This paper demonstrates an association between tolerance of violence against women and psychosocial distress experiences in humanitarian settings in northern Uganda. Our contribution is twofold. First, this paper confirms previous research that showed that psychosocial distress experiences are higher among refugees than host populations. Second, this paper shows that tolerance of violence against women aggravates psychosocial distress among refugees compared to host populations. However, tolerance of violence could be a coping mechanism for women experiencing violence at greater rates [54]. Being female, having a low education, and a low income are risk factors for experiencing psychosocial distress. These results indicate that income, and positive attitudes toward female-led income generating activities, act as protective measures against psychosocial distress [54]. These findings call for integrated programming to prevent and respond to violence against women, and mental health and psychosocial support. The findings reported in this paper also show that there is value in examining the intersections among violence against women and mental health and psychosocial distress, particularly in humanitarian settings. It is important to understand these intersections, in order to promote strategic and integrated programming that addresses risks that occur simultaneously, related to gender-based violence and psychosocial distress in humanitarian settings. Given the linkages between tolerance of violence against women and social norms, we recommend integrating social and gender norm changes in interventions that are aimed at preventing violence against women, and the associated psychosocial distresses in humanitarian settings, in northern Uganda.

## Figures and Tables

**Table 1:** The sample distribution across the study district by sex of the respondent.

Study District	Beneficiary Individuals	Actual Sample Size		Total Actual Sample Size
		Host	Refugee	
Adjumani	1140	43	180	223
Lamwo	615	74	144	218
Obongi	945	87	129	216
Total	2700	204	453	657

**Table 2:** Distribution of respondents by resident status.

Variable	Resident Status		Chi-Square (*p*-Value)
	Host	Refugee	
Age of respondent			11.1 ** (0.026)
15–24	8.8	11.7	
25–34	18.6	27.4	
35–44	21.6	17.0	
45–54	19.6	13.0	
55+	31.4	30.9	
Marital status			3.6 (0.162)
Currently married	82.9	74.7	
Divorced/separated/widowed	8.9	15.3	
Single	8.1	10.0	
Sex of respondent			1.6 (0.199)
Female	61.3	66.5	
Male	38.7	33.6	
Level of education			8.2 ** (0.041)
No school	26.0	34.9	
Primary	58.3	47.0	
Secondary	13.2	16.3	
Tertiary	2.5	1.8	
Personal income in the last one month			58.5 **** (0.000)
Less than UGX 10,000 (USD 2.7)	41.2	69.5	
UGX 10,000–20,000 (USD 2.7–USD 5.5)	28.9	20.8	
Above UGX 20,000 (USD 5.5)	29.9	9.7	
Women should participate in income generation			6.1 ** (0.013)
No	11.8	19.7	
Yes	88.2	80.4	
It is possible for men to stop violence			2.4 (0.299)
Agree	84.8	86.1	
Disagree	14.7	12.1	
Do not know	0.5	1.8	
Women are raped because of the way they dress			3.7 (0.156)
Agree	74.5	67.1	
Disagree	24.0	31.4	
Do not know	1.5	1.6	
Consent before sex is necessary			1.1 (0.591)
No	8.3	8.9	
Do not know	2.5	4.0	
Yes	89.2	87.2	
Women know where to obtain contraceptives			11.3 *** (0.004)
Yes	90.7	80.4	
No	5.4	13.0	
Do not know	3.9	6.6	
Women can decide the number of children to have			3.4 (0.180)
Yes	14.7	15.0	
No	84.8	82.3	
Do not know	0.5	2.7	
Study district			23.9 **** (0.000)
Adjumani	21.1	39.7	
Lamwo	36.3	31.8	
Obongi	42.6	28.5	
Tolerance of violence against women			1.1 (0.292)
No	38.2	42.6	
Yes	61.8	57.4	
Total (%)	100	100	
Total (*n*)	204	453	

Note: ** = *p* < 0.05; *** = *p* < 0.01; **** = *p* < 0.001. USD 1 is equivalent to UGX 365, as of 6 March 2021.

**Table 3:** Factors associated with the risk of experiencing psychosocial distress.

Variable	Model 1	Model 2
Tolerate violence (RC = No)		
Yes	1.07 (0.85–1.35)	6.86 ** (1.23–38.22)
Age of respondent (RC = 15–24)		
25–34		1.07 (0.44–2.64)
35–44		0.69 (0.28–1.73)
45–54		0.73 (0.27–1.95)
55+		2.50 (0.67–9.30)
Marital status (RC = Single)		
Married		1.74 (0.69–4.39)
Divorced/separated/widowed		-
Sex (RC = Male)		
Female		6.94 *** (1.76–27.32)
Level of education (RC = No education)		
Primary		4.73 ** (1.24–18.00)
Secondary		4.66 (0.96–22.56)
Tertiary		1.76 (0.40–7.74)
Personal income in last one month (RC = above UGX 20,000 (USD 5.5)		
Less than UGX 10,000 (USD 2.7)		3.37 ** (1.32–8.62)
UGX 10,000–20,000 (USD 2.7–USD 5.5)		2.16 (0.63–7.39)
Women should participate in income generation (RC = No)		
Yes		0.39 ** (0.17–0.89)
Women know where to obtain contraceptives (RC = Yes)		
No		0.51 (0.24–1.08)
Do not know		-
Study district (RC = Adjumani)		
Lamwo		1.61 (0.92–2.83)
Obongi		1.74 (0.88–3.44)
Resident status (RC = Host)		
Refugee		1.50 (0.93–2.42)
Interaction between sex and tolerant violence		
Female vs Yes		0.19 ** (0.04–0.82)
Interaction between level of education and tolerate violence		
Primary vs Yes		0.21 ** (0.04–0.96)
Secondary vs Yes		0.24 (0.04–1.42)
Tertiary vs Yes		0.11 (0.01–1.62)
Interaction between personal income and tolerate violence		
Less than UGX 10,000 (USD 2.7) vs Yes		0.45 (0.15–1.35)
UGX 10,000–20,000 (USD 2.7–USD 5.5) vs Yes		0.97 (0.24–4.00)
Constant	2.96 *** (2.49–3.52)	0.22 (0.03–1.46)
Number of observations	657	335
Likelihood ratio Chi-squared (probability)	0.38 (0.539)	60.55 (0.000)

Note: ** = *p* < 0.05; *** = *p* < 0.01. USD 1 is equivalent to UGX 3656 as of 6 March 2021. Confidence intervals in parentheses.

## Data Availability

The datasets used in this study are available from the corresponding author upon reasonable request.
